# Hydrodechlorination of Tetrachloromethane over Palladium Catalysts Supported on Mixed MgF_2_-MgO Carriers

**DOI:** 10.3390/molecules21121620

**Published:** 2016-11-25

**Authors:** Magdalena Bonarowska, Maria Wojciechowska, Maciej Zieliński, Angelika Kiderys, Michał Zieliński, Piotr Winiarek, Zbigniew Karpiński

**Affiliations:** 1 Institute of Physical Chemistry, Polish Academy of Sciences, Kasprzaka 44/52, Warsaw 01-224, Poland; mbonarowska@ichf.edu.pl (M.B.); mzielinski@ichf.edu.pl (M.Z.); 2 Faculty of Chemistry, Adam Mickiewicz University, Umultowska 89b, Poznań 61-614, Poland; emawoj@amu.edu.pl (M.W.); angelika.kiderys@gmail.com (A.K.); mardok@amu.edu.pl (M.Z.); 3 Faculty of Chemistry, Warsaw University of Technology, Noakowskiego 3, Warsaw 00-664, Poland; piotrw@ch.pw.edu.pl; 4 Faculty of Mathematics and Natural Sciences, School of Science, Cardinal Stefan Wyszyński University, Wóycickiego 1/3, Warsaw 01-938, Poland

**Keywords:** CCl_4_ hydrodechlorination, palladium, MgF_2_, MgO, MgO-MgF_2_, supports, Lewis acidity

## Abstract

Pd/MgO, Pd/MgF_2_ and Pd/MgO-MgF_2_ catalysts were investigated in the reaction of CCl_4_ hydrodechlorination. All the catalysts deactivated in time on stream, but the degree of deactivation varied from catalyst to catalyst. The MgF_2_-supported palladium with relatively large metal particles appeared the best catalyst, characterized by good activity and selectivity to C_2_-C_5_ hydrocarbons. Investigation of post-reaction catalyst samples allowed to find several details associated with the working state of hydrodechlorination catalysts. The role of support acidity was quite complex. On the one hand, a definite, although not very high Lewis acidity of MgF_2_ is beneficial for shaping high activity of palladium catalysts. The MgO-MgF_2_ support characterized by stronger Lewis acidity than MgF_2_ contributes to very good catalytic activity for a relatively long reaction period (~5 h) but subsequent neutralization of stronger acid centers (by coking) eliminates them from the catalyst. On the other hand, the role of acidity evolution, which takes place when basic supports (like MgO) are chlorided during HdCl reactions, is difficult to assess because different events associated with distribution of chlorided support species, leading to partial or even full blocking of the surface of palladium, which plays the role of active component in HdCl reactions.

## 1. Introduction

Catalytic hydrodehalogenation (HdCl) of harmful organic compounds, such chloromethanes, CFCs or chlorobenzenes on supported metal catalysts, appear a useful method for transforming those detrimental compounds into valuable chemicals [1,2]. The active phase of these catalysts are metals, among which the best performance show platinum, palladium and nickel [2,3,4,5]. Recently published review article by Colombo et al. [6] highlights the suitability of iron catalysts in removing chlorocarbons from the water. In addition, the performance of the iron in water decontamination can be greatly improved by doping with other metals such as palladium, copper, silver and nickel. Besides, the role of support should not only be considered as a metal redispersing medium. Carbon is the support of industrial choice for a number of reasons, including high specific surface area and stability against corrosion by halogen-containing products of hydrodehalogenation (HCl, HF). Research with the use of other supports, such as silica, alumina, zeolites or magnesia, served mainly for short-term model studies, when the functioning of various metallic phases is tested. The role of acido-basic properties of a support is not well understood, although several suggestions were made in this respect. Studied in the reaction of hydrodechlorination of 1,1-dichlorotetrafluoroethane, Pd catalysts supported on AlF_3_ and MgF_2_ (characterized by very dissimilar acidities) did not show large differences in their catalytic behavior [7]. It would speak against any special effect of support acidity on the hydrodechlorination performance. However, it is also known that the acidic or basic character of the support would modify metal particles, making them electrodeficient or electron-rich. The electron deficiency was found to depend on the concentration and the location of Brønsted acid sites [8]. Such effect would lead to a weaker interaction of the metal with reaction intermediates contributing to higher activities and product selectivities to, frequently desired, partly dehalogenated compounds [5,9]. Similarly, optimal combination of Pd^σ+^ and Pd° species has recently been invoked for the best hydrodechlorination performance [10,11]. Electrodeficient palladium, Pd^2+^, has been reported to improve the activity of Pd catalysts in HdCl reactions [12,13,14,15,16,17].

On the other hand, the presence of support acid centers would also lead to accumulation of polymeric species and catalyst’s fouling by coking [18,19]. Less acidic supports would act as chlorine “sink”, keeping the metal surface clean for a considerable time of reaction. This does not seem to function for highly acidic supports which, in addition, produce polymeric species, which in turn would be spilt over onto the metal surface [18].

In this respect, advantages of MgO as appropriate support of the hydrodechlorination catalyst are presented by several authors [20,21,22]. The high activity of the Pt/MgO catalyst was ascribed to basic properties of MgO. The basicity of this oxide seems to be responsible for retarding the coke formation and suppression of C_2_ oligomers (C_2_Cl_4_ and C_2_Cl_6_ mainly) which are the principal reasons for the catalyst deactivation. Clarke et al. [23] studied MgO-supported Pt catalysts in the hydrogenolysis of C_5_-C_6_ hydrocarbons and came to conclusion that large changes in the catalytic behavior of platinum result from negative charge from the magnesia O^2−^ ions to the metal.

Suitability of MgF_2_-supported metal catalysts for HdCl reactions was proved in numerous studies [7,14,20,21,24,25,26,27,28]. Because of a variety of investigated reactants, metal precursors (metal salts vs. phosphine complexes of metals, [28]), metal dispersion and structural forms of MgF_2_ (high surface area form [26] vs. well-crystalline [25]) it difficult to describe shortly this issue in much detail. However, a general overview appears to appreciate very good overall activity, selectivity to desired products and stability of MgF_2_-supported metals, usually exceeding the performance of their counterparts supported on oxidic carriers. The most often explanation takes into account the beneficial effect of MgF_2_ on metal centers making them electrodeficient. MgO → MgF_2_ transformation during HdCl of CFCs over MgO-supported metals was also considered as important in shaping the active form of metal catalysts [29].

We have decided to study the hydrodechlorination of CCl_4_ carried out over palladium catalysts supported on MgO-MgF_2_ carriers, in strong belief that the acido-basic properties of these mixed materials are well correlated with their composition. By increasing the fluorine content of the magnesium oxide fluoride, the Lewis acidity increases whereas the basicity decreases [30]. Therefore, these sites can be tuned over a wide range of MgO-MgF_2_ composition thus giving access to optimized catalytic activity and selectivity of these phases as was found for Michael additions [31]. However, during any hydrochlorination reactions which produce HCl the problem becomes more complicated. The reaction leads to a rapid transformation of MgO into MgCl_2_ [20,21], which has to be considered in description of a working state of the Pd/MgO-MgF_2_ catalyst. Full or even partial replacement of MgO by MgCl_2_ should also result in an increase in the strength of Lewis acid sites on the surface of the catalyst.

## 2. Results and Discussion

### 2.1. Catalyst Characterization

Characterization of prepared MgO-MgF_2_ carriers was reported in earlier publications, the “sol-gel” series in [32] and the “carbonate” series in [33] and are recalled in Table 1. For the “sol-gel” series, the content of magnesium oxide had a considerable effect on the surface area of the MgO-MgF_2_ systems, i.e., the surface area of MgO-MgF_2_ was seven times larger than that of pure magnesium fluoride and still larger than that of pure MgO. All the “sol-gel” samples were mesoporous with the mean pore diameters ranging from 8 to 18 nm. The size of the pores strongly depended on the composition of the samples and declined with increasing MgO content in MgO-MgF_2_ samples. For the “carbonate” series the surface area of the mixed MgO-MgF_2_ supports was 2–3 times greater than that of pure MgF_2_. In general, the surface area of mixed supports increased with increasing amount of MgO introduced. The BET surface area of MgO was almost five times greater than that of MgF_2_. All the “carbonate” series samples were mesoporous with the mean pore diameters ranging from 8 to 24 nm. The XRD studies showed the presence of separate crystalline MgO and MgF_2_ phases [33].

The present sample characterization concerns only the Pd-loaded MgO-MgF_2_ catalysts, with attention focused on the metal component which is commonly regarded as the active phase in hydrodechlorination reactions. Table 2 shows metal dispersion data based on H_2_ and CO chemisorptions. Considering the methodological (pulse vs. static) differences, the results obtained are internally consistent. In addition, the TPHD studies (Table 2) show larger H/Pd ratios for poorly dispersed palladium catalysts, in agreement with known correlations [34,35,36]. In the absence of XPS studies, these results would suggest that our palladium material reduced at 380 °C has not been significantly chemically modified by possible interactions with MgO-MgF_2_ supports.

However, it is found that introduction of a larger amount of palladium (3%) onto a support characterized by a smaller surface area and lower pore volume (here MgF_2_) results in the formation of relatively large Pd particles, i.e., ~8 nm (as assessed from CO chemisorption) compared to very small (~2 nm or less) metal particles deposited on MgO and MgO-MgF_2_ carriers. It appears that the support of less developed pore structure is not able accommodate larger amounts of dissolved metal precursor during impregation whereas a highly developed pore structure leads to evenly distributed small portions of the precursor. More important is that such large difference in metal dispersion makes a direct comparison of catalytic pattern in these series difficult. Fortunately, smaller differences in surface areas in the “carbonate” series of MgO-MgF_2_ supports and lower palladium loadings employed (1% instead of 3%) allowed to prepare the catalysts characterized by comparable metal dispersions (Table 2).

**Figure 1:**
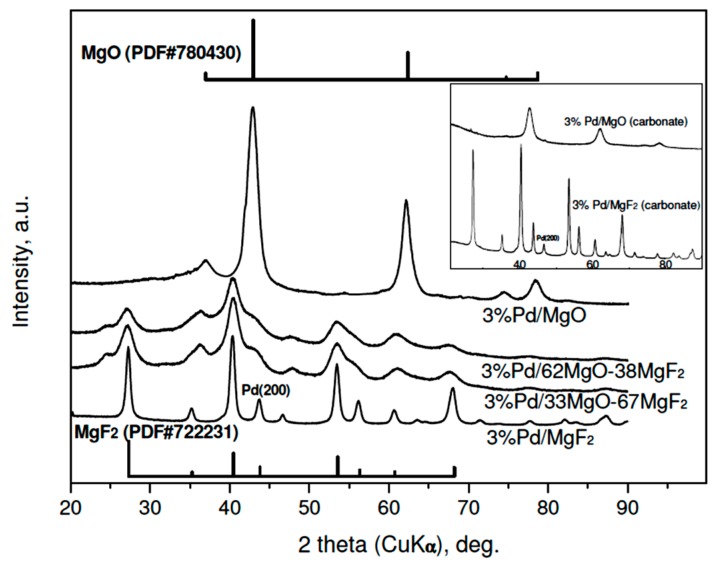
XRD profiles of reduced 3 wt % Pd/MgO-MgF_2_ catalysts (“sol-gel” series). Reflections for MgF_2_ and MgO are presented by bars proportional to their relative intensities. Inset: diffractograms of 3 wt % Pd catalysts of the “carbonate” series. To simplify the notation the composition of MgO-MgF_2_ mixture the % symbol is usually dropped in further text.

The results of XRD studies of reduced 3 wt % Pd/MgO-MgF_2_ catalysts are presented in Figure 1. A variety of large diffraction peaks from crystalline phases of MgO and MgF_2_, overlapping reflections from highly dispersed palladium, prevent identification of the metal. Only in the case of 3 wt % Pd/MgF catalysts, the (200) reflection from the Pd (fcc phase) of low intensity would be identified. Rough estimation of Pd crystallite size from that reflection (using the Scherrer formulae) gives 12–15 nm, i.e., the value in a limited agreement with the Pd particle sizes assessed from chemisorption (Table 1). Accordingly, the diffractograms of the less metal loaded (1 wt %) Pd/MgO-MgF_2_ catalysts resemble very much the profiles of unloaded supports [33], although a very small (200) reflection from palladium was also detected in the profile of the 1 wt % Pd/MgF_2_ (XRD data not shown).

### 2.2. Catalytic Performance of Pd/MgO-MgF_2_ in CCl_4_ Hydrodechlorination

#### 2.2.1. Catalytic Activity

All tested Pd/MgO-MgF_2_ catalysts deactivated during the hydrodechlorination of CCl_4_ at 90 °C, but the character of deactivation varied between different samples.

**Figure 2:**
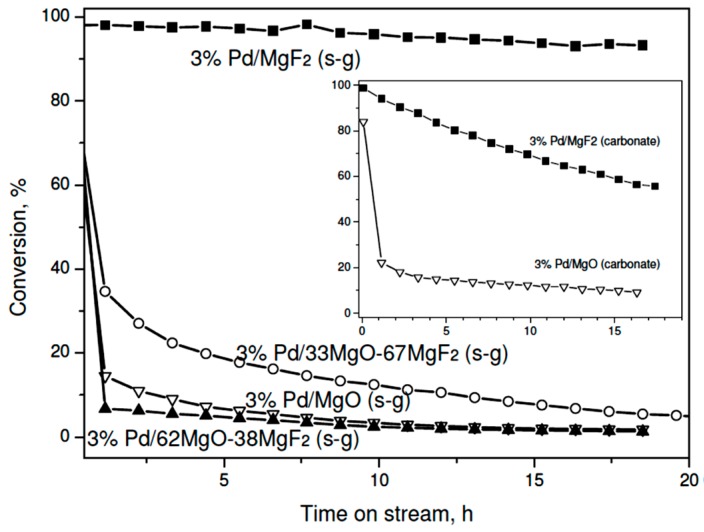
Time on stream behavior of 3 wt % Pd/MgO-MgF_2_ (sol-gel) catalysts in CCl_4_ hydrodechlorination at 90 °C. Inset: The behavior of the “carbonate” series of catalysts.

Reasons for deactivation of HdCl metal catalysts were analyzed in previous publications. They are considered as metal surface chloriding by a liberated HCl [38,39,40,41], massive deposition of carbonaceous deposits on metal surface [42,43,44,45] and metal sintering caused by long-term operation [46]. It was rather well established that smaller Pd particles (~2 nm or less) deactivate faster than larger Pd crystalllites (~10 nm in size) [36]. Accordingly, our magnesia-containing Pd samples in the sol-gel series which are characterized by very small metal particles (Table 2) showed very pronounced deactivation during kinetic runs and low final activity (Figure 2). On the other hand, the superior catalytic performance, with a small decrease of conversion (from ~100% to ~93%) showed the 3 wt. % Pd/MgF_2_ catalysts (sol-gel series), characterized by poor metal dispersion. Its counterpart from the carbonate series also exhibited high conversion, but a marked deactivation was observed in this case (from ~100% to ~56%, Figure 2, inset). Such somewhat different “deactivation” behavior is understood when one considers that the former catalyst was screened at near full conversion level, i.e. the amount of catalyst sample was too large (always ~0.4 g). However, the turnover frequencies for both catalysts at final conversions (after 15–16 h of reaction) are similar (Table 3), so the apparent difference in the behavior of two 3 wt % Pd/MgO catalysts seen in Figure 2 is easily understood in terms of different metal particle sizes (7.9 vs. 15.6 nm), which differ by factor of ~2. For this metal particle range (~10 nm) one should not expect any special structure-sensitivity, which was usually observed for metal dispersions between 0.2 and 1 [47]. It must be emphasized that the final activity of MgF_2_-supported Pd catalysts was higher than that previously obtained for Pd/Al_2_O_3_ [48] and active carbon-supported palladium [49,50] catalysts. This effect would speak for the beneficial effect of support acidity in electronic modification of palladium surface (creation of Pd^σ+^ species).

**Figure 3:**
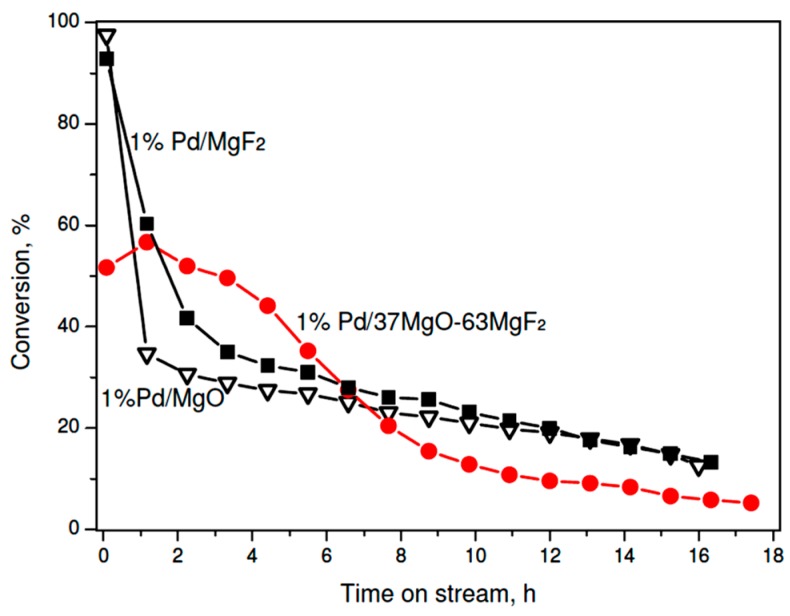
Time on stream behavior of 1 wt % Pd/MgO-MgF_2_ (carbonate) catalysts in CCl_4_ hydrodechlorination at 90 °C.

On the other hand, very different palladium dispersions in the sol-gel series prevent any speculation about a possible role of support in shaping the catalytic behavior. Therefore, it is concluded that palladium particle size of palladium, which is commonly considered as the catalytically active phase, must be a dominant factor. Thus, to investigate a possible role of support one needs to test palladium catalysts characterized by a similar metal dispersion. As mentioned earlier preparation of the carbonate series of 1 wt % Pd/MgO-MgF_2_ catalysts, gave the catalysts characterized by similar metal particle sizes (~4 nm) (Table 2). Now the MgF_2_-supported catalyst showed a poorer performance than its counterparts characterized by 3 wt % metal loading (Figure 3). Again, such effect would be ascribed to the metal particle size effect. Unexpectedly, the activity time on stream behavior of 1 wt % Pd/MgO was similar to that shown by 1 wt % Pd/MgF_2_, although larger differences in product selectivity were found (next subsection). On the other hand, the MgO-MgF_2_ supported catalyst showed an interesting behavior: For a longer time on stream the overall activity was higher and more stable than that for Pd/MgO and Pd/MgF_2_. This effect will be discussed in the last part of discussion.

#### 2.2.2. Product Selectivities and Activation Energies

We consider that chlorine-free compounds (i.e., hydrocarbons) are the desired products of CCl_4_ hydrodechlorination because chloroform, a previously desired product, is classified as an extremely hazardous substance. In this respect all tested here MgF_2_-supported palladium catalysts showed the best results, as indicated in Table 3, where one column aggregates C_2_-C_5_ hydrocarbon products. A higher activation energy for MgO-supported palladium would suggest that the removal of surface chlorine (as HCl) is more difficult than for the MgF_2_-supported metal. This comparison clearly confirms the suitability of MgF_2_-supported metal catalysts for HdCl reactions [14,20,21,24,25,26].

#### 2.2.3. Working State of MgO-MgF_2_ Supported Palladium Catalyst

**Figure 4:**
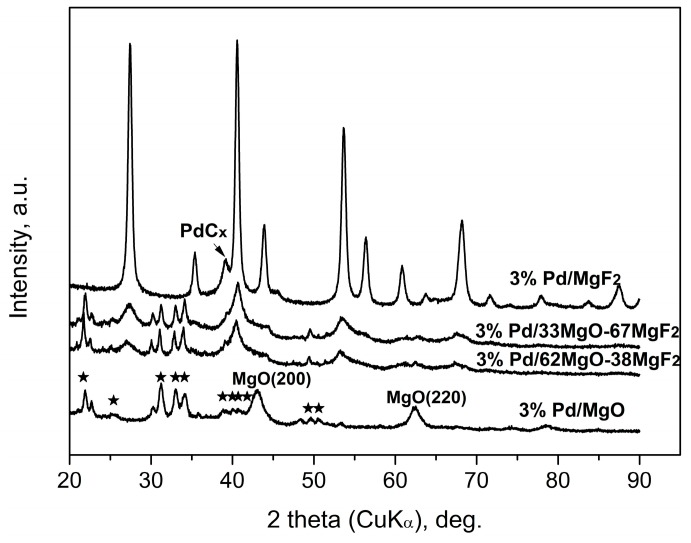
XRD profiles of 3 wt % Pd/MgO-MgF_2_(sol-gel) catalysts after CCl_4_ hydrodechlorination. The five-pointed stars show the main reflections of MgCl_2_∙6H_2_O (PDF #760789).

**Figure 5:**
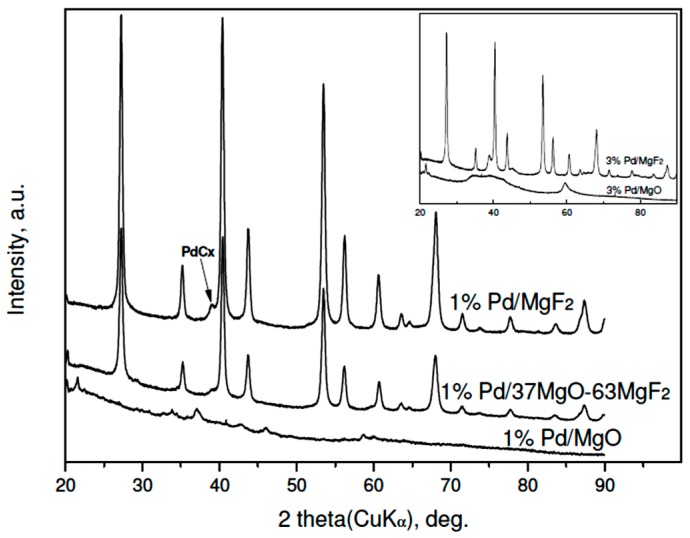
XRD profiles of Pd/MgO-MgF_2_(carbonate) catalysts after CCl_4_ hydrodechlorination.

**Figure 6:**
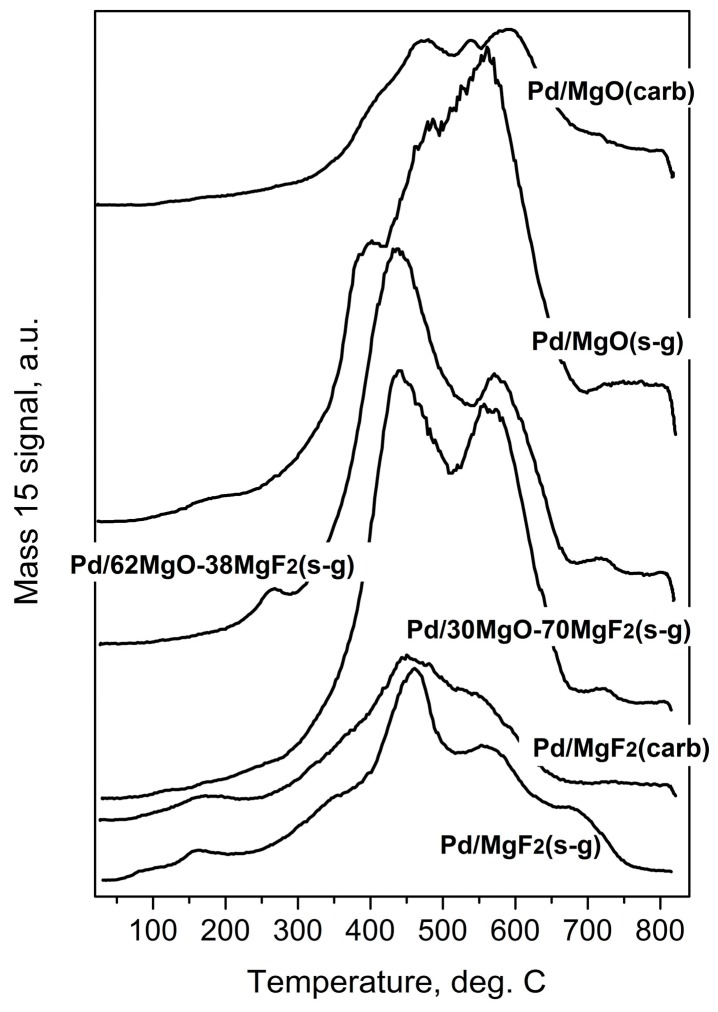
Temperature programmed hydrogenation of post-reaction deposits from Pd/MgO-MgF_2_ catalysts. Mass 15 was selected for monitoring methane evolution because mass 16 would also reflect the signal from water.

**Figure 7:**
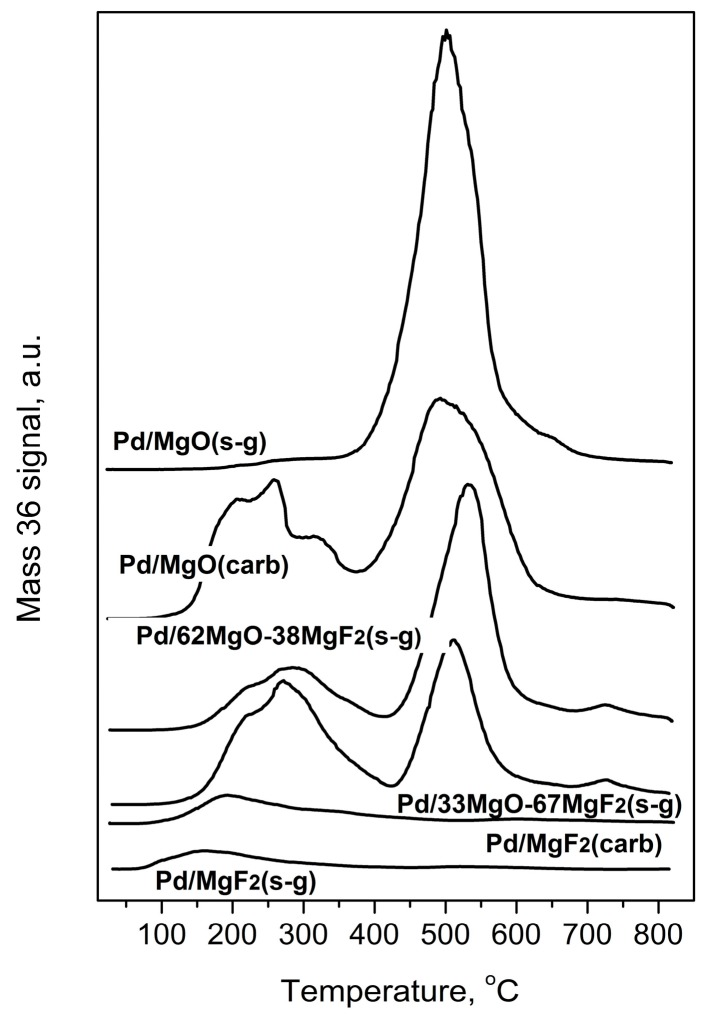
Evolution of HCl during Temperature Programmed Hydrogenation of post-reaction deposits from Pd/MgO-MgF_2_ catalysts.

**Figure 8:**
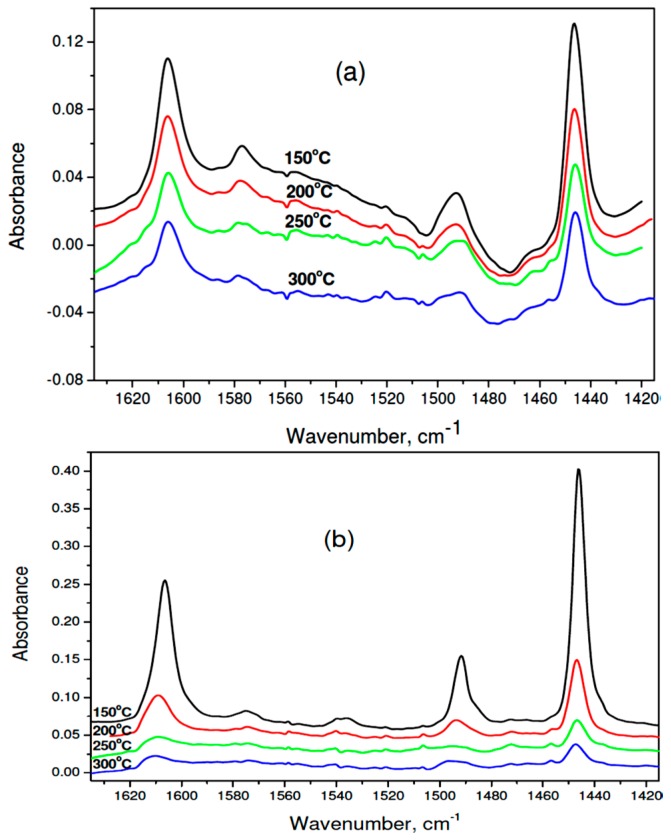
IR spectra of adsorbed pyridine on 1 wt % Pd/37MgO-63MgF_2_ catalyst: (**a**) after reduction, (**b**) after CCl_4_ hydrodechlorination. Numbers ascribed to spectra indicate the temperature of pyridine desorption.

The used samples of catalysts were investigated by XRD, temperature programmed hydrogenation of post-reaction deposits and IR spectroscopy of adsorbed pyridine (on selected samples). The results shown in Figure 4, Figure 5, Figure 6, Figure 7 and Figure 8 and Table 4 allow to make cautious speculations about the working state of HdCl catalysts.

X-ray results (Figure 4 and Figure 5) indicate that palladium, when it is detectable in the diffractograms, is largely transformed into a PdC_x_ phase. Such transformation is often observed for HdCl reactions carried out over Pd catalysts [49,51] and here confirmed for MgF_2_-supported Pd catalyst used in the CCl_4_ reaction. This phenomenon may decide about the time scale behavior of product selectivity pattern because at an initial period of reaction palladium acts as carbon sink decreasing the selectivity to methane [47,51].

Figure 4 and Figure 5 showed big changes for the magnesium-containing support component. Direct transformation of MgO into MgCl_2_ is also not unexpected, as a similar effect was observed for Pt/MgO catalyst by Choi et al. [20]. However, a closer inspection of diffractograms allows estimation of the extent of transformation and, hopefully, the state of crystallinity of formed MgCl_2_. Figure 4 shows that magnesia in 3 wt % Pd/MgO catalyst is only partly transformed into MgCl_2_. In MgO-MgF_2_ supported 3% Pd loaded catalysts the extent of transformation appears more marked. Figure 5 shows analogous data for the carbonate series of Pd catalysts. Here the degree of MgO → MgCl_2_ transformation is still more pronounced. For the 1 wt % loaded catalysts the presence of MgCl_2_ and MgO phases is only slightly marked (for 1 wt % Pd/MgO) or even completely invisible for 1 wt % Pd/37MgO-63MgF_2_ catalyst. These observations along with much broader reflections from the MgCl_2_ in the 3 wt % Pd/MgO catalyst that the formed MgCl_2_ phase has a tendency to spread out over the catalyst surface.

Temperature programed hydrogenation of post-reaction deposits presented in Figure 6 and Figure 7 shows that all tested catalysts possess different amounts of carbon and chlorine. MgF_2_-supported catalysts are relatively deprived of chlorine: only some small amount of HCl is liberated at low temperature (≤200 °C), suggesting reduction of chloride associated with the palladium. A nonappearance of high temperature HCl evolution (observed for MgO-containing catalysts) indicates lack of chlorine on the surface of MgF_2_ (Figure 7). This, in turn, suggests no changes in the acidity of this catalyst which would be associated with chlorine. However, CH_4_ evolution from this catalyst was quite pronounced (Figure 6) but still not as high as for other catalysts, which we believe is a primary reason of catalyst deactivation in HdCl reactions. Interestingly, Figure 7 shows no chlorine liberation for Pd/MgO at a low temperature region. We believe that it is not due to a lack of chlorine on palladium surface but rather because the surface of palladium is effectively blocked by an impermeable coating by MgCl_2_, [52] or simply closed in a porous structure by MgCl_2_ “stoppers”. As it was suggested earlier, a decreased crystallinity of MgCl_2_ in this catalyst allows assumption that MgCl_2_ is either spread out over the support or a topochemical reaction MgO-MgCl_2_ [52] would lead to dramatic changes in the support texture, with possible burial of Pd particles. The MgO-MgF_2_ supported catalysts showed both the low and high temperature HCl evolution, evidencing that both MgCl_2_ spreading as well as textural changes would be less dramatic for mixed MgO-MgF_2_ systems, where a part of palladium species is deposited on MgF_2_. It looks like the very huge HCl evolution from Pd/MgO occurred at very high temperature largely results from reduction of MgCl_2_.

The results of IR spectra of adsorbed pyridine (Figure 8a,b and Table 4) allow to confirm the presence of Lewis acid centers on the surface of all tested catalysts. The analysis of population and strength distribution of these centers was carried out by considering the absorbance of 1446 cm^−1^ band, associated with pyridine coordinated to the Lewis acid center. The largest amounts of pyridine left on the surface after desorption at 150 °C were found for used 1 wt % Pd/37MgO-67MgF_2_ catalyst. The number of such centers was nearly three times higher than the respective value for the reduced catalyst. However, these centers are rather weak because after desorption at 200 °C only 28.5% of them still bind pyridine. On the other hand, at analogous conditions, as far as 66% of pyridine is left on the reduced catalyst. A quantitative parameter characterizing the strength of acid centers is the ratio of the 1446 cm^−1^ absorbance after pyridine desorption at 300 and 200 °C. Table 4 displays that the highest value of this parameter (0.64) showed the reduced 1 wt % Pd/37MgO-67MgF_2_ catalyst, whereas after HdCl it decreased to 0.26. A similar value was found for 1 wt % Pd/MgO after reaction (0.25). Therefore, it appears that during the HdCl reaction the strongest acid centers are neutralized with simultaneous formation of weak acid centers. It would be worthwile to investigate the acid strength evolution in the course of reaction and combine it with the catalytic performance (activity and selectivity).

In summary, the role of support acidity is quite complex. On the one hand, a definite, although not very high Lewis acidity of MgF_2_ is beneficial for shaping high activity of palladium catalysts. The MgO-MgF_2_ support characterized by stronger Lewis acidity than MgF_2_ contributes to very good catalytic activity for a relatively long reaction period (~5 h) but subsequent neutralization of stronger acid centers (by coking) eliminates them from the catalytic amphitheater. On the other hand, the role of acidity evolution, which takes place when basic supports (like MgO) are chlorided during HdCl reactions, is difficult to assess because different events associated with distribution of chlorided support species, leading to partial or even full blocking of the surface of palladium, which plays the role of active phase in HdCl reactions. Therefore, a unique disposal of straightforward tuneability of acido-basic properties of MgO-MgF_2_ of varied composition is weakened by the interaction with chloride species. It must recalled that these sites, tuned over a wide range of MgO-MgF_2_ composition, gave an unproblematic access to optimized catalytic activity and selectivity of these phases for the Michael addition of 2-methylcyclohexane-1,3-dione to methyl vinyl ketone [31]. By increasing the fluorine content of the magnesium oxide fluoride, the Lewis acidity increases whereas the basicity decreases [30]. This correlation is also compatible with recent results on toluene hydrogenation over Ir/MgO-MgF_2_ catalysts [53], where the best iridium catalysts were supported on MgF_2_-rich carriers characterized by a relatively low surface basicity.

## 3. Experimental Section

### 3.1. Preparation of MgO-MgF_2_ Supports

Two series of MgO-MgF_2_ supports were prepared. The first one was synthesized by the sol-gel method starting from magnesium methoxide treated with an aqueous solution of hydrofluoric acid, as described in [32]. The amount of HF solution was adjusted to obtain 40 and 70 mol % MgF_2_ in the samples. The resulting dense gels of MgO-MgF_2_ were subjected to ageing for 40 h at RT, and then to drying at 80 °C for 3 h. The dried samples of this “sol-gel” series were calcined for 4 h at 400 °C and sieved (selected fraction 0.25–0.5 mm). The MgF_2_ support was obtained by the sol-gel method from Mg(OCH_3_)_2_ and anhydrous HF (48.8% HF in methanol, Sigma-Aldrich, Polska, Poznań, Poland) in a way analogous to the above described synthesis for MgO-MgF_2_, but under rigorously anhydrous conditions. MgO was obtained by the sol-gel method by hydrolysis of magnesium methoxide (120 cm^3^ of 0.5 M solution) in water and treated similarly to MgO-MgF_2_. Another series of MgO-MgF_2_ supports of different MgF_2_ contents (0, 60% and 100%) were obtained in the reaction of basic magnesium carbonate (4MgCO_3_·Mg(OH)_2_·5H_2_O) powder with controlled amounts of 40 wt % aqueous solution of HF, as described in [33]. The resulting dense gels of this “carbonate” series were aged for 40 h at room temperature under stirring, followed by drying at 80 °C for 24 h and calcining under air flow at 500 °C for 4 h. The MgO support was obtained by decomposition of 4MgCO_3_·Mg(OH)_2_·5H_2_O at 500 °C for 4 h.

### 3.2. Preparation of MgO-MgF_2_ Supported Palladium Catalysts

The 3 wt % Pd/MgO-MgF_2_ catalysts were prepared by impregnation of both series of MgO-MgF_2_ supports with an acetone solution of palladium acetate using the incipient wetness technique. The 1 wt % Pd-loaded catalysts were prepared by wet impregnation of the “carbonate” supports with a methanol solution of palladium acetate. After impregnation and drying (at 80 °C for 20 h), the resulting solids were transferred to glass-stoppered bottles and kept in a desiccator. Prior to chemisorption and reaction studies, all catalysts were reduced in flowing 20% H_2_/Ar (50 cm^3^/min), ramping the temperature from 20 to 380 °C (at 8 °C/min), and kept at 380 °C for 2 h.

### 3.3. Characterization of Catalysts

The catalysts were characterized by H_2_ and CO chemisorption, XRD, temperature-programmed methods and IR spectroscopy of adsorbed pyridine. Irreversible uptake of hydrogen was measured in a pulse method system at 70 °C, to avoid the formation of β-PdH. CO adsorption was measured at 35 °C in a static system, using a double isotherm method (ASAP 2020 from Micromeritics). H_ad_/Pd and CO_ad_/Pd ratios were taken as measures of metal dispersion. Prior to characterization, the catalysts were pretreated in the same way as before kinetic experiments (next subsection), i.e., with the final reduction at 380 °C for 2 h. Reduced and post-reaction catalysts were investigated by X-ray diffractometry (PANalytical Empyrean, Almelo, The Netherlands, with Ni-filtered CuK_α_ radiation and sample spinner).

After H_2_ chemisorption, the samples were cooled to room temperature and subjected to a temperature programmed study in 10% H_2_/Ar flow, ramping the temperature from 20 to 150 °C, at 8 °C/min. Since the samples had already been reduced, the aim of such experiments was to monitor hydrogen evolution during decomposition of Pd hydride, in the temperature programmed hydride decomposition (TPHD). For details, see [34]. Post-reaction catalyst samples were investigated by temperature programmed hydrogenation (TPH-MS). TPH-MS runs were carried out in a flowing 10% H_2_/He mixture (25 cm^3^/min) at a 10 °C/min ramp and followed by mass spectrometry (MA200 Dycor-Ametek, Pittsburgh, PA, USA). For details, see [49].

The FTIR spectra of adsorbed pyridine were collected using a Thermo Scientific Nicolet 6700 (Madison, WI, USA) equipped with a MTC-A detector in transmission mode with a spectral resolution 4 cm^−1^. The OMNIC software (version 7.3, Thermo Electron Corporation, Madison, WI, USA) was used to collect and analyze spectra of adsorbed pyridine on selected (mainly post-reaction) catalysts samples which, after crushing, were pressed into self-supporting pellets of 18 mm diameter and mass ~20 mg. Prior to adsorption the sample was outgassed at 350 °C down to 10^−5^ mbar for 5 h. Then, it was cooled to room temperature and the IR spectrum was collected. Next, the sample was heated to 150 °C and contacted with pyridine vapor (dried over KOH, distilled and outgassed) for 10 min and pyridine desorption was carried out for 30 min. After collecting the spectrum, the temperature was gradually raised by 50 °C. In this way spectra of adsorbed pyridine at 200, 250 and 300 °C were collected and analyzed after subtracting background measured before pyridine adsorption.

### 3.4. Hydrodechlorination of Tetrachloromethane

Prior to the reaction, the catalyst charge (∼0.4 g) was dried at 120 °C for 0.5 h in an argon flow and reduced in flowing 20% H_2_/Ar (25 cm^3^/min), ramping the temperature from 120 °C to 380 °C (at 8 °C/min) and kept at 380 °C for 2 h. The reaction of HdCl of tetrachloromethane was carried out at 90 °C, the H_2_:CCl_4_ ratio ∼14:1 and total flow 29 cm^3^/min, in a glass flow system, as previously described [50]. The partial pressures of the reaction mixture were: CCl_4_ 4.3 kPa, H_2_ 60.5 kPa, Ar 36.5 kPa. The reaction (at 90 °C) was followed by gas chromatography. After catalyst screening at 90 °C and reaching a nearly constant conversion, the temperature was gradually decreased to 80 °C and 70 °C, and new experimental points were collected. Finally, the reactor was heated to 90 °C, and, in nearly all cases, the previous results collected at 90 °C were restored. A typical run lasted∼20 h.

## Figures and Tables

**Table 1:** Support characterization (adapted from [32,33]).

Support	BET Surface Area (m^2^·g^−1^)	Average Pore Diameter (nm)	Pore Volume (cm^3^·g^−1^)
**Sol-Gel Series**			
MgO	152	10.5	0.39
62% MgO-38% MgF_2_	208	8.2	0.43
33% MgO-67% MgF_2_	144	17.8	0.64
MgF_2_	32	15.5	0.12
**Carbonate Series**			
MgO	142.7	8.19	0.29
37% MgO-63% MgF_2_	51.0	23.79	0.30
MgF_2_	32.0	21.26	0.17

**Table 2:** Characteristics of Pd/MgO-MgF_2_ catalysts: palladium dispersion and H/Pd ratio from Temperature Programmed Hydride Decomposition (TPHD).

Support Series ^a^	Catalyst	H_ad_/Pd From Pulse Chemisorption and Metal Particle Size (d, nm) ^b^	CO_ad_/Pd From Static Chemisorption and Metal Particle Size (d, nm) ^c^	H/Pd From TPHD
sol-gel	3% Pd/MgO	0.509 (2.2)	0.661 (1.7)	0.12
	3% Pd/62% MgO-38% MgF_2_	0.495 (2.3)	0.645 (1.7)	0.13
	3% Pd/33% MgO-67% MgF_2_	0.554 (2.0)	0.672 (1.7)	0.11
	3% Pd/MgF_2_	0.175 (6.4)	0.141 (7.9)	0.30
carbonate	3% Pd/MgO	0.647 (1.7)	0.633 (1.8)	0.11
	3% Pd/MgF_2_	0.098 (11.4)	0.072 (15.6)	0.35
carbonate	1% Pd/MgO	n.m. ^d^	0.323 (3.5)	n.m. ^d^
	1% Pd/37% MgO-63% MgF_2_	n.m. ^d^	0.317 (3.5)	n.m. ^d^
	1% Pd/MgF_2_	n.m. ^d^	0.259 (4.3)	n.m. ^d^

^a^ For support preparation and description, see subsection 3.1. ^b^ From pulse chemisorption of H_2_ at 70 °C. Numbers in the parentheses indicate mean sizes of palladium particles calculated from the formula d_nm_ = 1.12/(H_ad_/Pd) [37]. ^c^ From static chemisorption of CO at 35 °C, taken for TOF calculation. Numbers in the parentheses indicate mean sizes of palladium particles calculated from the formula d_nm_ = 1.12/(CO_ad_/Pd). ^d^ Not measured.

**Table 3:** CCl_4_ hydrodechlorination on Pd/MgO-MgF_2_ catalysts. Final conversions, product selectivities, TOFs and activation energies.

Catalyst	Final Conversion ^a^	Product Distribution ^a^, %						TOF ^b^, s^−1^	Activation Energy ^c^, kJ/mol
		CH_4_	C_2_-C_5_	CH_x_Cl_4x_	C_2_H_x_Cl_y_	C_3_H_x_Cl_y_	ΣC_1_-C_5_		
**Sol-Gel Series**									
3% Pd/MgO	1.4	20.9	15.5	3.7	52.9	7.0	36.4	1.95 × 10^−4^	56.7 ± 1.4
3% Pd/62% MgO-38% MgF_2_	1.8	17.4	19.9	11.4	43.8	7.5	37.3	2.38 × 10^−4^	50.0 ± 2.1
3% Pd/33% MgO-67% MgF_2_	4.9	16.1	23.3	8.9	47.8	3.9	39.4	6.10 × 10^−4^	51.3 ± 5.2
3% Pd/MgF_2_	93.4	20.5	42.2	8.8	24.5	4.0	62.7	5.55 × 10^−2^	38.3 ± 1.8
**Carbonate Series**									
3% Pd/MgO	9.5	16.9	25.9	11.8	38.8	6.6	42.8	1.57 × 10^−3^	48.5 ± 4.6
3% Pd/MgF_2_	56.1	23.9	35.1	15.9	20.5	4.6	59.0	5.36 × 10^−2^	42.1 ± 1.3
1% Pd/MgO	13.7	17.0	10.6	7.8	62.9	1.6	27.7	2.15 × 10^−2^	65.0 ± 6.2
1% Pd/37% MgO-63% MgF_2_	5.5	26.6	22.6	3.5	40.8	6.5	49.2	5.08 × 10^−3^	50.7 ± 5.6
1% Pd/MgF_2_	14.1	20.6	32.9	15.9	26.3	4.3	53.5	1.51 × 10^−2^	46.3 ± 5.7

^a^ Final conversions and product selectivities after 15–16 h of time on stream (reaction temperature 90 °C); ^b^ Turnover frequency (at 90 °C) after 15–16 h of time on stream, based on dispersion data shown in Table 2 (CO/Pd); ^c^ Based on overall conversions collected at 70, 80 and 90 °C.

**Table 4:** Desorption of pyridine from 1 wt% Pd/MgO-MgF_2_, 1 wt % Pd/MgF_2_ and 3 wt % Pd/MgO catalysts. Absorbance (A) of 1446 cm^−1^ band (LPy) after desorption at different temperatures.

Catalyst Sample	T_des_, °C	A	%A	A_300_/A_200_
1 % Pd/37MgO-63MgF_2_ reduced	150	0.124	100.00	
	200	0.082	66.13	0.644
	250	0.059	47.58	
	300	0.0528	42.58	
1 % Pd/37MgO-63MgF_2_ after HdCl	150	0.337	100.00	
	200	0.096	28.49	0.339
	250	0.0325	9.64	
	300	0.0254	7.54	
1 % Pd/MgF_2_ after HdCl	150	0.0874	100.00	
	200	0.0414	47.37	0.360
	250	0.0237	27.12	
	300	0.0149	17.05	
3% Pd/MgO after HdCl	150	0.0049	100.00	
	200	0.0045	91.84	0.251
	250	0.00127	25.92	
	300	0.00113	23.06	
